# Regulatory RNA at the root of animals: dynamic expression of developmental lincRNAs in the calcisponge *Sycon ciliatum*

**DOI:** 10.1098/rspb.2015.1746

**Published:** 2015-12-22

**Authors:** Jon Bråte, Marcin Adamski, Ralf S. Neumann, Kamran Shalchian-Tabrizi, Maja Adamska

**Affiliations:** 1 Centre for Epigenetics, Development and Evolution (CEDE), Department of Biosciences, University of Oslo, Oslo, Norway; 2 Sars International Centre for Marine Molecular Biology, University of Bergen, Bergen, Norway; 3 Research School of Biology, Australian National University, Canberra, Australian Capital Territory, Australia

**Keywords:** lncRNA, lincRNA, regulatory RNA, sponges, development, evolution

## Abstract

Long non-coding RNAs (lncRNAs) play important regulatory roles during animal development, and it has been hypothesized that an RNA-based gene regulation was important for the evolution of developmental complexity in animals. However, most studies of lncRNA gene regulation have been performed using model animal species, and very little is known about this type of gene regulation in non-bilaterians. We have therefore analysed RNA-Seq data derived from a comprehensive set of embryogenesis stages in the calcareous sponge *Sycon ciliatum* and identified hundreds of developmentally expressed intergenic lncRNAs (lincRNAs) in this species. *In situ* hybridization of selected lincRNAs revealed dynamic spatial and temporal expression during embryonic development. More than 600 lincRNAs constitute integral parts of differentially expressed gene modules, which also contain known developmental regulatory genes, e.g. transcription factors and signalling molecules. This study provides insights into the non-coding gene repertoire of one of the earliest evolved animal lineages, and suggests that RNA-based gene regulation was probably present in the last common ancestor of animals.

## 1. Introduction

Long non-coding RNAs (lncRNAs) are usually defined as RNA transcripts which are several hundred nucleotides long but have no obvious protein-coding potential, although, in some cases, they might be translated, yielding short peptides of unknown function [1,2]. lncRNAs can regulate the expression of other genes through a variety of different mechanisms. The gene regulatory power of lncRNAs lies in their ability to interact with DNA in a site-specific manner, and at the same time bind to different proteins, bridging chromosomes and protein complexes [1,3–5]. Most nuclear lncRNAs function by guiding chromatin modifying proteins to specific genomic positions and can sometimes organize entire chromosomes or epigenetically alter chromosome states [6–8]. On the other hand, cytoplasmic lncRNAs regulate translation and stability of coding transcripts as well as protein localization (reviewed in [9]).

The lncRNA category comprises a wide variety of RNA transcripts, including both polyadenylated and non-polyadenylated lncRNAs that may be sense or antisense, intronic and intergenic with respect to protein-coding genes [10]. However, most studies on lncRNAs focus on polyadenylated lncRNAs that do not overlap other protein-coding genes, the so-called long ‘intervening’, or ‘intergenic’, non-coding RNAs (lincRNAs; [11]). lincRNAs seem to be expressed in a more tissue-specific and developmental stage-specific manner than protein-coding genes; in fact, embryonic development seems to be a very active time for lincRNA expression [12–15].

The action of lincRNAs during development has mostly been investigated in model vertebrate species. In zebrafish, a large number of lincRNAs are expressed during embryogenesis [16], and developmental regulatory functions have been demonstrated for two lincRNAs tested in knock-down and rescue by overexpression experiments [11]. In mice, more than a thousand lincRNAs are differentially expressed during postnatal testis development [17], and many lincRNAs are essential for survival and correct brain development [18]. Developmental lncRNAs have also been identified among invertebrates, for example in the nematode *Caenorhabditis elegans* [19], and recently Gaiti *et al.* [15] described dynamically expressed lncRNAs across multiple developmental stages of the demosponge *Amphimedon queenslandica*.

It has been hypothesized that an RNA-based gene regulation was important for the evolution of increased developmental complexity in animals [1]. However, it is currently not known whether this mode of gene regulation is exclusive to bilaterian animals, or whether this ‘hidden layer’ of gene regulation was already present in the earliest evolved (i.e. non-bilaterian) animal lineages. The findings of Gaiti *et al.* [15] based on embryonic and postembryonic development of *A. queenslandica* suggest that the latter scenario is correct. However, whether this is a general phenomenon among sponges (or other non-bilaterians) is still unknown.

Therefore, the aim of this study was to identify lincRNAs expressed during embryonic development in the calcisponge *Sycon ciliatum* (Calcaronea), a representative of one of the earliest evolved animal lineages. We have taken advantage of the existing large-scale RNA-Seq data [20] and systematically searched for long non-coding transcripts in different stages of embryogenesis. We identify 2421 transcribed lincRNAs and *in situ* hybridization (ISH) of selected representatives confirms that calcisponge lincRNAs are specifically and dynamically expressed in embryonic and somatic cells. More than 600 lincRNAs are specifically upregulated during embryogenesis. Finally, we have identified co-expressed modules of lincRNAs and coding genes that are active during specific stages of embryonic development and which are enriched for development-related functional categories. This study provides, to our knowledge, the first insight into the non-coding repertoire of calcisponges and supports the notion that RNA-based gene regulation was already present in the last common ancestor of all animals.

## 2. Methods

### (a) Transcriptome assembly and identification of lincRNAs

*Sycon ciliatum* genome and protein-coding focused transcriptome assemblies have been previously described [20,21]. In this work, we have reassembled the transcriptome de novo from non-strand-specific poly(A)+ RNA-Seq reads using Trinity and detected protein-coding regions with Transdecoder with default parameters [22]. We chose de novo assembly over genome-driven assembly to alleviate effects of allelic variation between the genome and transcriptome (derived from different specimens) on on-genome alignment. Such variation influences on-genome alignment of short reads in much greater level than alignments of already assembled (and thus longer) transcripts. There were 46 967 unique transcripts identified as protein coding (minimum open reading frame (ORF) length 300 bp) and 46 824 as long non-coding (minimum length 600 bp; this stringent cut-off has been implemented to allow potential testing of expression by ISH in subsequent steps). The transcripts were aligned on the *S. ciliatum* genome assembly with Exonerate [23], which identified the structures of 26 349 coding and 21 680 non-coding genes. To ensure that the non-coding transcripts are truly not of coding origin (e.g. pseudogenes or remnants of retrotransposon activity), the 46 824 non-coding transcripts were used as queries in a BlastX search [24] against the NCBI RefSeq protein database (http://www.ncbi.nlm.nih.gov/refseq/). The Blast output was parsed with the BlastGrabber program [25], and sequences that gave a hit with an e-value of less than 10 were discarded. Such conservative e-value was chosen to ensure that no transcript of possibly coding origin was retained. The retained transcripts were translated in all six reading frames using transeq of the EMBOSS package [26] and used as queries in a HMMER search (e-value cut-off 0.01; [27]) against the PfamA database [28], as well as an additional Blastp search against the NCBI RefSeq database (e-value cut-off 10). The remaining transcripts were evaluated for protein-coding potential using the coding potential calculator [29]. All sequences with a coding potential score larger than 1 were discarded. In total, this left 10 548 transcripts from 6856 different genes that were putatively termed lncRNAs. As our assemblies are based on non-strand-specific libraries, differentiation between natural antisense transcripts and misassembled fragments of protein-coding genes is difficult. We have thus removed all sequences overlapping ORFs and introns of coding genes, leaving a dataset of 2421 intergenic lncRNAs (lincRNAs) for further analysis.

### (b) *In situ* hybridization

To select candidate lincRNAs for ISH analysis, we used criteria which, in our hands, routinely give highly specific and robust expression patterns: expression level at least 40 counts in at least one library combined with at least 20-fold expression difference between any two stages. Of the 209 sequences satisfying these criteria, we have manually selected four transcripts representing diverse expression profiles (unique to early embryonic stages; peaking in the larvae; expressed throughout embryogenesis with or without expression in the larvae). Eight hundred to one thousand nucleotide fragments were amplified by PCR for each lincRNA and cloned using the pGEM-T easy vector system II (Promega, USA). Digoxigenin-UTP-labelled RNA probes were synthesized in both directions with SP6 and T7 RNA polymerases (Roche, USA) and cleaned using the RNeasy MinElute cleanup kit (Qiagen, USA). *Sycon ciliatum* specimens were collected in fjords near Bergen, Norway (N 60°27′33″, W 4°56′1″) between May and July 2013. The specimens were fixed, stored, hybridized and photographed as described in [30].

### (c) Identification of independently regulated lincRNAs

To select lincRNAs with independently regulated expression, all lincRNAs with expression correlated with the nearest protein-coding gene neighbour (either upstream or downstream of the lincRNA) were discarded. Expression profiles of all identified coding and lincRNA genes across a range of developmental stages were calculated with use of the RSEM package [31] as described previously [20]. The neighbouring pairs were identified using closest-features in the BEDOPS toolkit v. 2.4.3 [32]. The Spearman correlation between pairs of a lincRNA and its neighbour gene was calculated in R v.3.1.2 [33], and *p*-values were corrected for multiple comparisons with the Benjamini–Hochberg (BH) procedure [34]. lincRNAs with a strong expression correlation with a protein-coding neighbour were discarded (*ρ* ≥ 0.6, BH-adjusted *p*-value <0.05). Principal-components analysis (PCA) was performed with DESeq2 [35] on log-transformed normalized counts (using DESeq2 regularized log transformation). Differential expression (DE) tests were performed using DESeq2 (Wald test with BH-adjusted *p*-values <0.1).

### (d) Identification of co-expressed modules of lincRNAs and coding genes

To focus the analysis on relevant genes and to reduce the computational load, we only included the 10 560 coding genes which are differentially upregulated in any developmental stage compared with non-reproductive tissue, in addition to the 1853 identified lincRNAs. Furthermore, we discarded coding genes and lincRNAs with low variance between developmental stages; we required normalized counts higher than five in three or more samples, and we used only lincRNAs and coding genes with an expressional variance in the top 75% (variance calculated based on log-transformed (log_2_(*x* + 1) normalized counts). This filtering left 2615 transcripts (2421 coding genes and 194 lincRNAs). The module identification was done using the R package WGCNA v. 1.41 [36]. Modules were identified using the ‘dynamic topological overlap matrix’-method and requiring a minimum module size of 30 (see the WGCNA manual). Briefly, Pearson correlations were calculated between all pairs and converted into an adjacency matrix using a power function (soft thresholding power 18). Adjacencies were converted into topological overlaps and clustered by hierarchical clustering in R. Modules were defined as branches cut-off using the dynamicTreeCut algorithm in WGCNA. Modules were assigned colour labels, which were then converted to letters from A-W (see the electronic supplementary material, figure S2).

### (e) lncRNA blast search

The longest isoform from each of the 6856 lncRNA loci (repeats masked by Tandem Repeats Finder [37] and Repeat Masker [38]) was Blast searched (blastn word size 4, e-value cut-off 1 × 10^−4^, minimum query overlap 25%) against the genomes of *Ciona intestinalis*, *Hydra magnipapillata*, *Nematostella vectensis*, *Amphimedon queenslandica*, *Oscarella carmela*, *Pleurobrachia bachei*, *Mnemiopsis leidyi*, *Trichoplax adhaerens*, *Salpingoeca rosetta*, *Sphaeroforma arctica* and *Capsaspora owczarzaki*, as well as the recently published *A. queenslandica* lncRNAs [15].

### (f) Gene ontology analysis

All coding transcripts were searched for homologues against NCBI Refseq using BlastX. Blast results were imported into Blast2GO [39] and combined with conserved protein domain detection using InterProScan in Blast2GO to generate a gene ontology. In total, 10 552 genes were annotated. GO-enrichments of the different co-expressed modules were analysed by Ontologizer ([40]; topology-weighted method and *p*-value cut-off of 0.05). The GO-enrichment results were inspected manually and also visualized using the Enrichment Map Cytoscape plugin [41].

## 3. Results and discussion

### (a) Thousands of lincRNAs are dynamically transcribed in *Sycon ciliatum*

**Figure 1.**
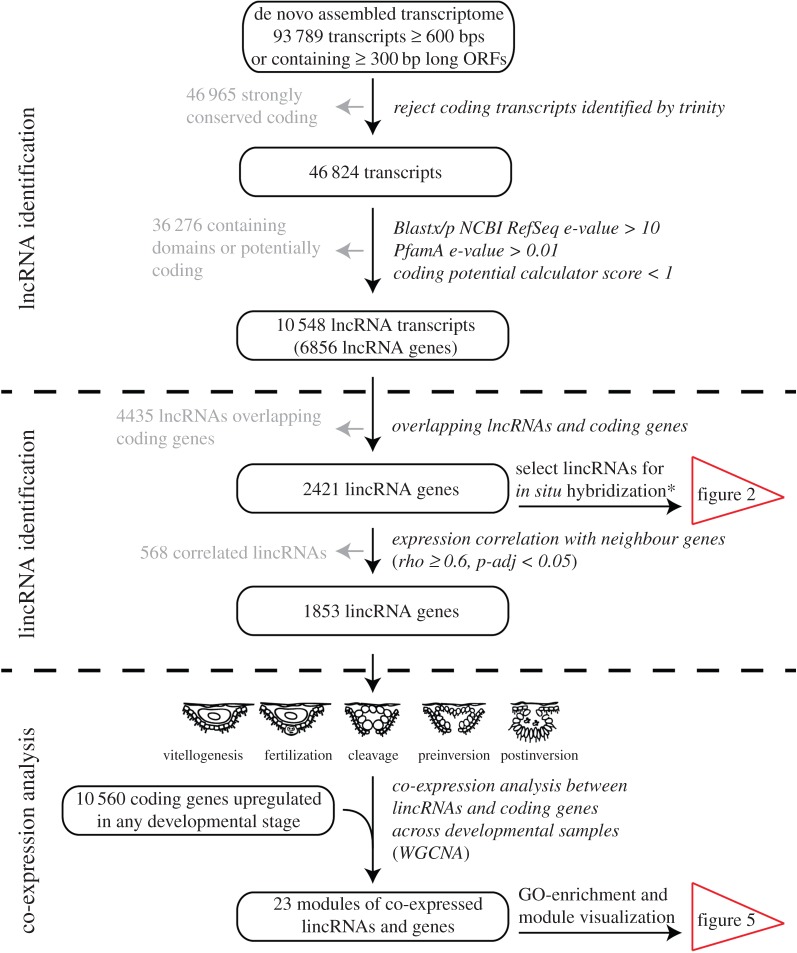
Overview of the filtering pipeline to detect lincRNAs in *Syon ciliatum*. The starting point of the analysis was a transcriptome assembled de novo from non-strand-specific pair-end RNA-Seq data (see the Methods section for details). Asterisk (*): criteria for selecting lincRNAs for *in situ* hybridization were expression level of at least 40 counts in at least one library combined with minimum 20-fold expression difference between any two developmental stages.

An outline of the procedure aimed at identification of lincRNAs potentially involved in development of the calcareous sponge *S. ciliatum* is presented in figure 1. In the first step, we have used previously described non-strand-specific RNA-Seq datasets [20,21] to re-assemble the transcriptome, including non-coding sequences (our previous pipeline was focused on discovery of ORFs) and map it to the genome. A combination of Blast searches against reference databases, protein domain searches and ORF evaluation resulted in annotation of 2421 non-coding loci identified as putative lincRNAs.

**Figure 2.**
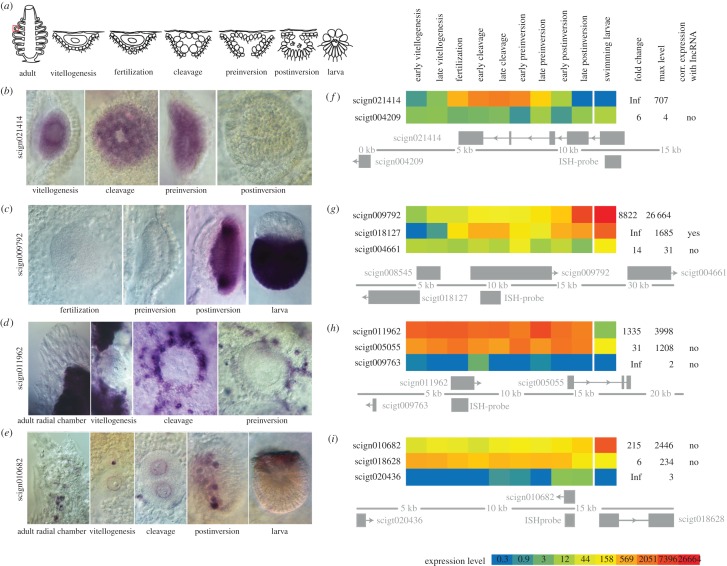
*In situ* hybridization (ISH) detection of developmentally expressed lincRNAs. (*a*) Overview of the different developmental stages used for the ISH and selection of lincRNAs for ISH. (*b*–*e*) ISH expression patterns of the lincRNAs during developmental stages. (*f*–*i*) Heatmap representation of expression of investigated lincRNAs and their nearest protein-coding neighbours on the genome, as well as a representation of the genomic localization. Significant correlations require *ρ* ≥ 0.6 and *p*-value <0.05 (Spearman correlation).

Similar to what has been found in other studies, the lincRNAs were generally shorter than coding genes, with the majority of transcripts being below 1000 nts (electronic supplementary material, figure S1). In addition, the majority of lincRNAs were unspliced (i.e. single-exon transcripts), although a large number also contained multiple exons. To find out whether these genes might be developmentally regulated, both in terms of temporal and spatial expression, we have used a combination of bioinformatics and ISH. For the *in silico* part of the protocol, RNA-Seq libraries representing all key stages of oogenesis and embryonic development: vitellogenesis, fertilization, cleavage, cell differentiation and morphogenesis (referred to as preinversion and postinversion stages in calcaronean sponges) embedded in the maternal tissue, as well as free swimming larvae were used (figure 2*a*). We have visualized expression profiles of genes which, based on our experience in the *Sycon* model system, were likely to be robustly detected if studied by ISH (see Materials and methods). Among the 2421 queried putative lincRNAs genes, we selected four representatives with different developmental expression patterns for the subsequent ISH analysis. Consistent with the RNA-Seq data analysis, detection of all four probes revealed specific and unique expression patterns (figure 2*b–e*). In particular, the expression of scign021414 was limited to early stages of embryonic development and detected only in the embryonic cells until the preinversion stage, but not in surrounding maternal tissues (figure 2*b*). By contrast, scign009792 was not detectable in the oocytes or early embryos, but displayed strong expression in postinversion stage and larval micromeres (figure 2*c*). The remaining two genes were expressed in maternal cells only, or in both maternal and embryonic cells. scign011962 was detected in a variable fraction of choanocytes, especially those surrounding the oocytes and embryos, but not in the oocytes or embryos themselves (figure 2*d*). Finally, scign010682 was detected in a small number of unidentified small somatic cells, oocytes and early cleavage blastomeres (where it displayed nuclear and perinuclear localization), maternal cells ingressing into larval cavity during postinversion and in larval macromeres (figure 2*e*). Notably, in all cases, labelling was detected from one strand only, indicating unidirectional expression of all of the four lincRNAs studied by ISH. Thus, it appears that as in bilaterians, calcisponge lincRNAs display a striking variety of expression patterns, encompassing all embryonic cell types as well as multiple somatic cell types. In addition, their expression is clearly restricted to specific cell types and time points during development, which indicates that they are subjected to a tightly regulated transcriptional control.

### (b) Hundreds of lincRNAs with independently regulated developmental expression

In bilaterian model systems, lncRNAs are often co-expressed with their coding genomic neighbours, which they sometimes overlap [42,43]. We have investigated genomic locations and expression of surrounding genes for the four selected examples of lincRNAs (figure 2*f*–*i*). As in the case of the expression patterns, the relationships between the position and expression of lincRNAs and their neighbours were varied. Three of the lincRNAs displayed no correlation of expression with their coding neighbours (figure 2*f,g*,*i*). Interestingly, the expression of scign009702 was moderately correlated (*ρ* = 0.68, *p* < 0.001) with scigt018127 transcribed in the opposite direction (figure 2*g*).

Given the diversity of the expression profiles and genomic organization of the large number of lincRNAs reflected by the four ‘case studies’ described above, we decided to systematically investigate lincRNA embryonic expression and co-expression with coding genes. To avoid artefacts caused by misassembly (such as misassembled fragments of UTRs) or erroneous transcription, and co-expression driven by genomic proximity rather than by functional relationships, we chose to focus this part of analysis on the lincRNAs with expression regulated independently of their neighbouring coding genes. We tested the 2421 lincRNAs for correlated expression with their closest protein-coding gene both upstream and downstream. Five hundred and sixty-eight lincRNAs were moderately or strongly correlated with a protein-coding neighbour (*ρ* ≥ 0.6, BH-adjusted *p*-value < 0.05) and were discarded. Altogether, this left 1853 lincRNAs that we further analysed for potential association with development.

For this analysis, we have used the RNA-Seq libraries from the embryogenesis series for which biological replicates were available, as well as samples of sponges collected outside of the reproductive season and not containing any discernible oocytes or embryos [20]. Only mid-body slices of both reproductive and non-reproductive sponges were used as the oscular (apical) region of *S. ciliatum* has a different transcriptional makeup (as shown previously [20]).

**Figure 3.**
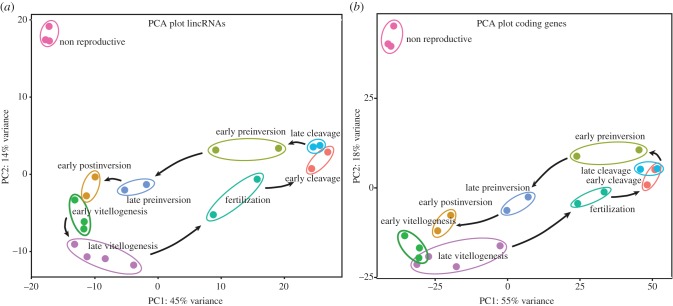
Principal-components analysis (PCA) plot. Plotting of the PCA on rlog-transformed gene expression counts from the different developmental stages of *Sycon ciliatum* of (*a*) lincRNAs and (*b*) coding genes. The analysis was done on the 500 most variable genes and the samples are plotted on their first two principal components. Each dot represents RNA-Seq data of a developmental sample.

We first wanted to know whether the expression of lincRNAs was structured according to the developmental stages and if so, whether this structure was similar or different to that of the coding genes. PCA demonstrated that expression of the lincRNAs was indeed strongly structured according to the different developmental stages, with non-reproductive tissue distant from all the other stages (figure 3*a*). This result is inline with our expectation that different pools of transcripts are active during the different stages of development. Notably, the structuring of lincRNAs expression seems to be very similar to that of the coding genes (figure 3*b*), and thus lincRNAs are likely to be involved in development similarly to coding genes.

**Figure 4.**
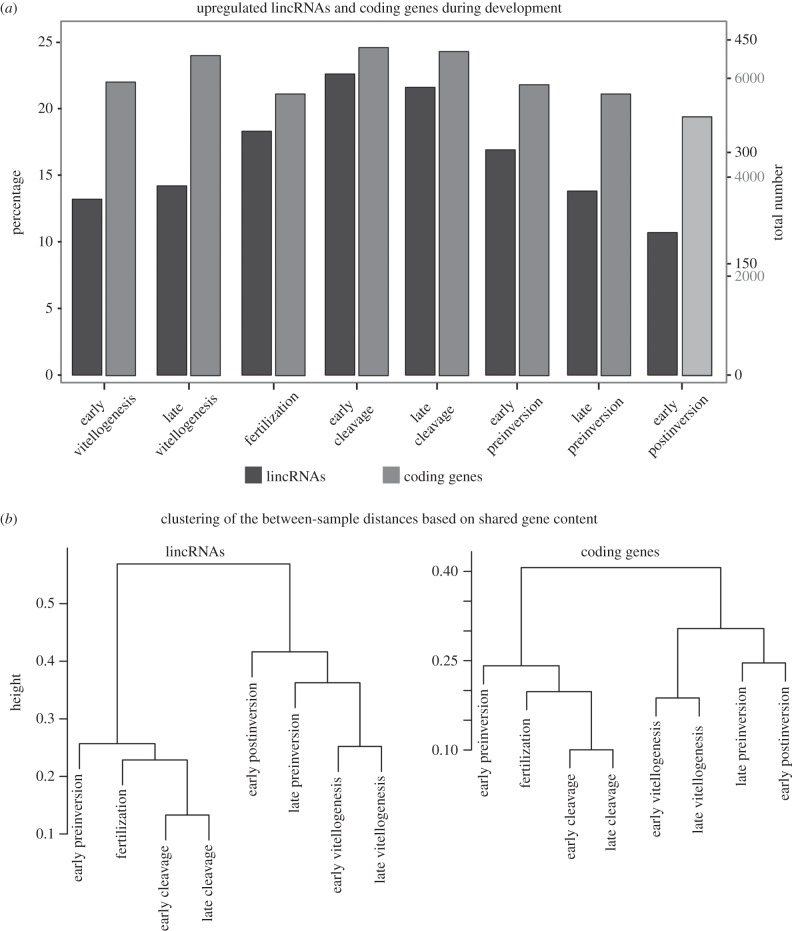
Differentially expressed lincRNAs and coding genes during development. (*a*) Histograms showing the number of significantly upregulated lincRNAs and coding genes between non-reproductive stages and reproductive stages. (*b*) Hierarchical clustering of the distances between developmental samples calculated on the basis of the shared number of upregulated lincRNAs or coding genes.

To identify lincRNAs that were significantly upregulated during any of the developmental stages, we then tested for genes differentially expressed between non-reproductive samples and each developmental stage separately (figure 4). In total, 622 lincRNAs (33.6% of all independently regulated lincRNAs) were significantly upregulated in at least one of the developmental stages compared with non-reproductive tissue. In virtually all of the developmental stages, more than 200 lincRNAs were upregulated (except for 198 in early postinversion), with the cleavage stages displaying the highest numbers of upregulated lincRNAs (419 and 400). Successive stages of the development share the majority of upregulated lincRNAs, and 85 lincRNAs are upregulated across all developmental stages. On the other hand, only a small number of lincRNAs are uniquely upregulated in any developmental stage, with the highest number of unique lincRNAs (32) found during early cleavage. Thus, the cleavage stages appear to represent a period of very active transcription of a diverse pool of lincRNAs, perhaps in preparation to embryonic cell differentiation which will be occurring during subsequent developmental stages.

### (c) lincRNAs are integral components of co-expressed gene modules including developmental regulatory genes

**Figure 5.**
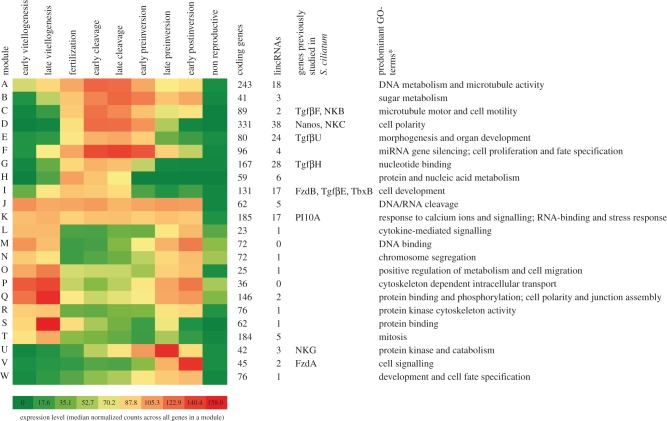
Overview of the co-expressed modules. The modules of co-expressed coding genes and lincRNAs are named from A to W. The heatmap is generated based on the median normalized expression values of all genes in a module. Asterisk (*): the predominant GO-terms are named on the basis of the major clusters of GO-terms in each module identified by the Enrichment Map analysis. In cases where no clusters were identified, the Ontologizer results were inspected manually.

Gene regulatory networks and modules are central for the control and timing of organismal development. However, little is known whether non-coding genes are expressed in such modules. We therefore sought to identify modules of co-expressed coding genes and lincRNAs active during embryonic development. The co-expression analysis resulted in identification of 23 different modules (named A-W), with 21 of these including one or more lincRNA (figure 5 and the electronic supplementary material, figure S2). Two modules were almost uniformly expressed (indicated in figure 5 as the median of the normalized expression counts across all genes in a module) across development (J and K), and a few modules were restricted to a very narrow window during development, such as modules U and W only active during the latest stages of embryonic development (preinversion and postinversion).

The other modules showed two main patterns of expression; a large fraction of the modules seems to have the highest expression from fertilization to late cleavage or early preinversion stages (A–I). On the other hand, several modules (e.g. modules L–R) displayed a biphasic expression pattern almost opposite to modules A–I, with one peak during vitellogenesis and a second peak during morphogenesis stages (late preinversion and early preinversion). Given the fact that the second wave of oogenesis in *S. ciliatum* overlaps with these developmental stages, it is unclear whether this profile of expression is owing to expression in oocytes undergoing oogenesis only, or to expression present in oocytes, decreasing during cleavage and increased again in late preinversion stage embryos. Similar patterns of expression of protein-coding genes have indeed been previously observed, for example in the case of *SciBcatA* [20], although this gene is also strongly expressed in the somatic cells (choanocytes) of the adult tissue, and as such has not been recovered in our dataset of developmentally upregulated genes.

On the other hand, eight of the identified modules (but none of the strongly ‘biphasic’ modules) included protein-coding developmental genes (esp. components of the *Wnt* and *TgfBeta* pathways and transcription factors) with extensively studied expression patterns in *S. ciliatum* [20,21]. In addition, GO-term enrichment analysis (figure 5) indicated that several of the modules were particularly rich in terms related to developmental processes. For example, module I, including *SciFzdB*, *SciTGFBE* and *SciTbxB*, was particularly enriched in terms related to cell development and transcription factors. Module D, which included coding genes such as *SciNanos* and *SciNKC*, contained many genes related to cell differentiation and development, tissue and organ development and transcription regulation. Genes of both of these modules had a peak of expression during cleavage; with module D genes having a narrower peak of expression than module I. Similarly, a high fraction of genes included in module E (containing for example also *SciTGFBU*) have functions related to morphogenesis and organ development.

### (d) lncRNAs as regulatory elements of animal development

It is becoming evident that lncRNAs are important for correct development of many animal lineages, for instance in mouse [18], zebrafish [11,16] and *C. elegans* [19]. Recently, lncRNAs expressed during development were also identified in the demosponge *A. queenslandica* [15]. In this study, we provide a first glimpse into the rich repertoire of regulatory RNAs involved in embryonic development of another early branching animal, the calcisponge *S. ciliatum*. This has important evolutionary implications; first of all, it suggests that using regulatory RNAs during early development is an ancestral feature of all sponges. Second, as both sponges and other animal lineages express lncRNAs during development, this feature was probably present already in the last common animal ancestor.

However, an important question is whether all sponges and other animals use homologues lncRNAs during development, or if they have acquired different types of lncRNAs during evolution. We did not identify any conserved lncRNAs between *S. ciliatum* and any other metazoan or non-metazoan opisthokont species. The lack of sequence similarity between lncRNAs across animal phyla (conserved lncRNAs have so far only been detected between vertebrate species [11,44]) suggests that these lncRNAs belong to different families, supporting the latter scenario. However, they could still have conserved secondary and tertiary structures, and thereby conserved function, despite being highly diverged on the primary sequence level.

The uncertain evolutionary history and the few functional studies undertaken so far makes it difficult to study lncRNA roles in an evolutionary developmental framework. One way to overcome this problem is to identify conserved modules, or networks, of co-expressed genes including lncRNAs. One such example could be the developmental lncRNAs co-expressed with *Frizzled B* (a key component of the Wnt-pathway) in both *A. queenslandica* [15] and *S. ciliatum* in this study (module I; figure 5).

Another challenge is that the availability of developmental transcriptome series is phylogenetically very patchy. Therefore, there is a need for high-quality staged transcriptome data from other deep-branching animal lineages, including ctenophores and placozoans. Such datasets might allow us to test whether, although lncRNAs are not conserved at the primary sequence level, they operate in deeply conserved gene regulatory networks.

Altogether, our work demonstrates that lncRNA expression during calisponge development is highly dynamic with restricted temporal and spatial patterns. Although it is uncertain whether these lncRNAs are homologous to those in other animals, the use of long non-coding RNAs in embryonic development is probably an ancestral feature of all animals.

## Supplementary Material

Distribution of transcript lengths and exon numbers.

## Supplementary Material

Co-expressed module detection

## Supplementary Material

GO-enrichment analysis of selected modules

## Data accessibility

The following datasets are freely accessible on the Dryad Digital Repository (https://datadryad.org; doi:10.5061/dryad.v83fj): list of lincRNAs, PCR-primers used for ISH probe synthesis, coding and non-coding gene models, gene expression data, lists of co-expressed modules and the main R-commands used in this study.
