# Supplementary figures and images for: Regulatory RNA at the root of animals: dynamic expression of developmental lincRNAs in the calcisponge Sycon ciliatum
Source: Proc Biol Sci. 2015 Dec 22;282(1821):20151746. doi: 10.1098/rspb.2015.1746 (PMC4707743; doi:10.1098/rspb.2015.1746)

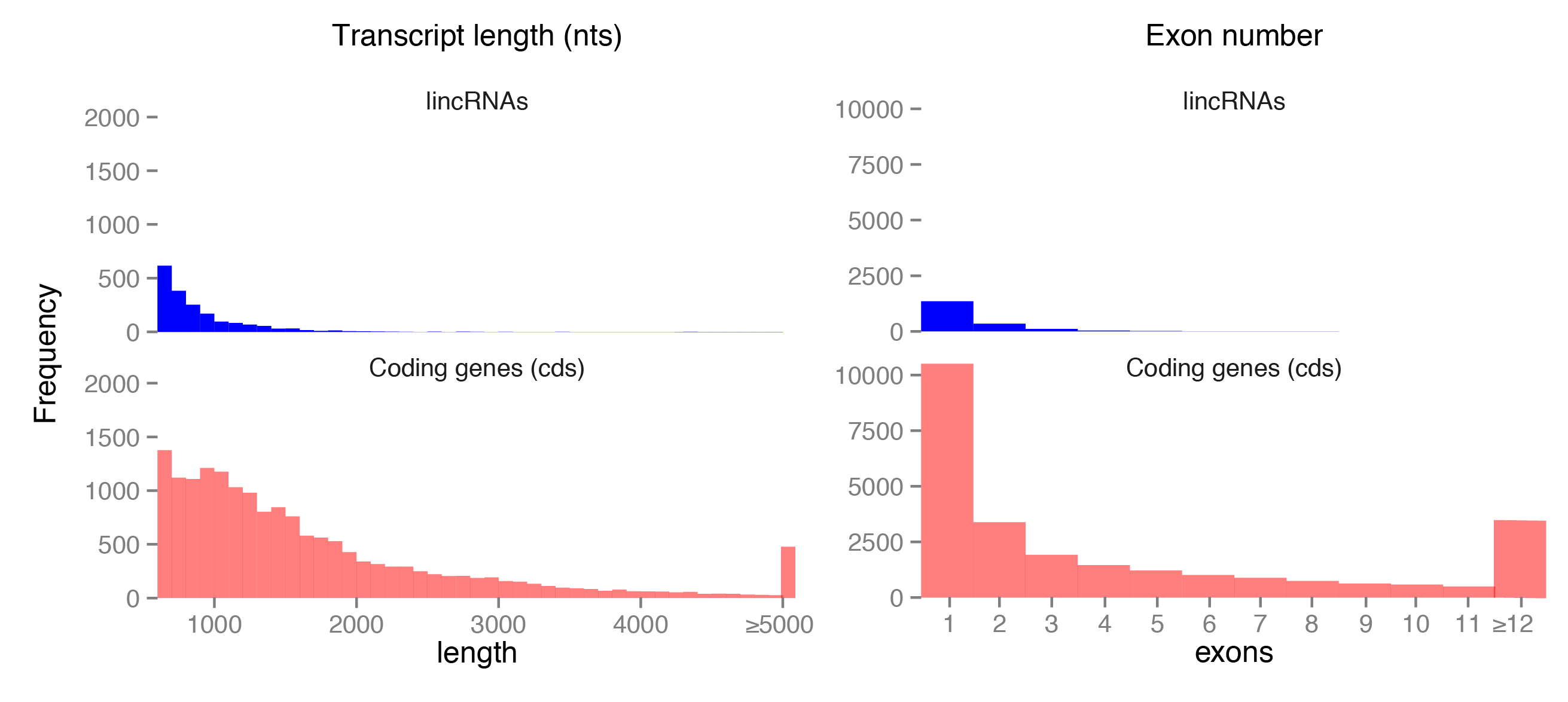

Supplement: Distribution of transcript lengths and exon numbers. [file rspb20151746supp1.png]

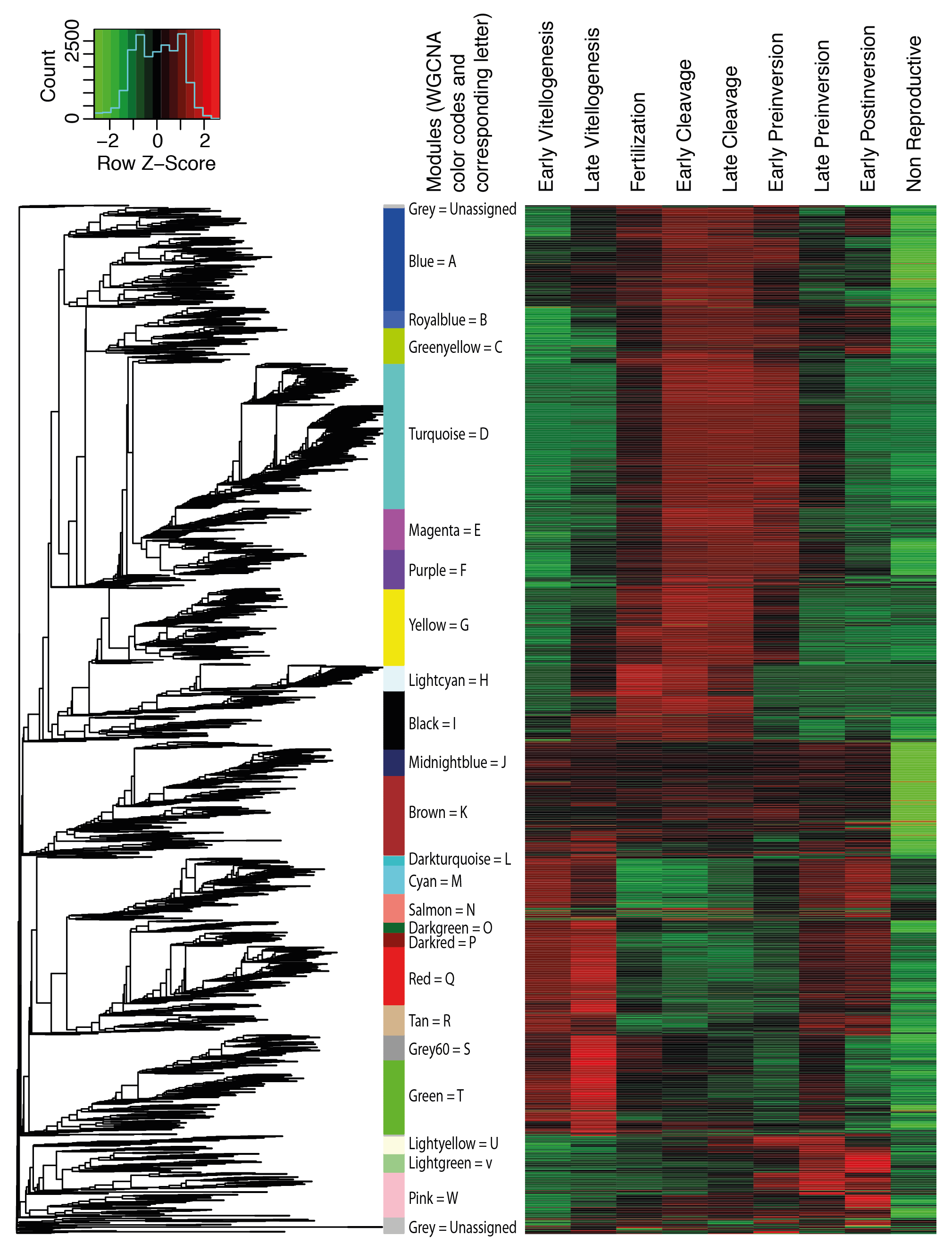

Supplement: Co-expressed module detection [file rspb20151746supp2.png]

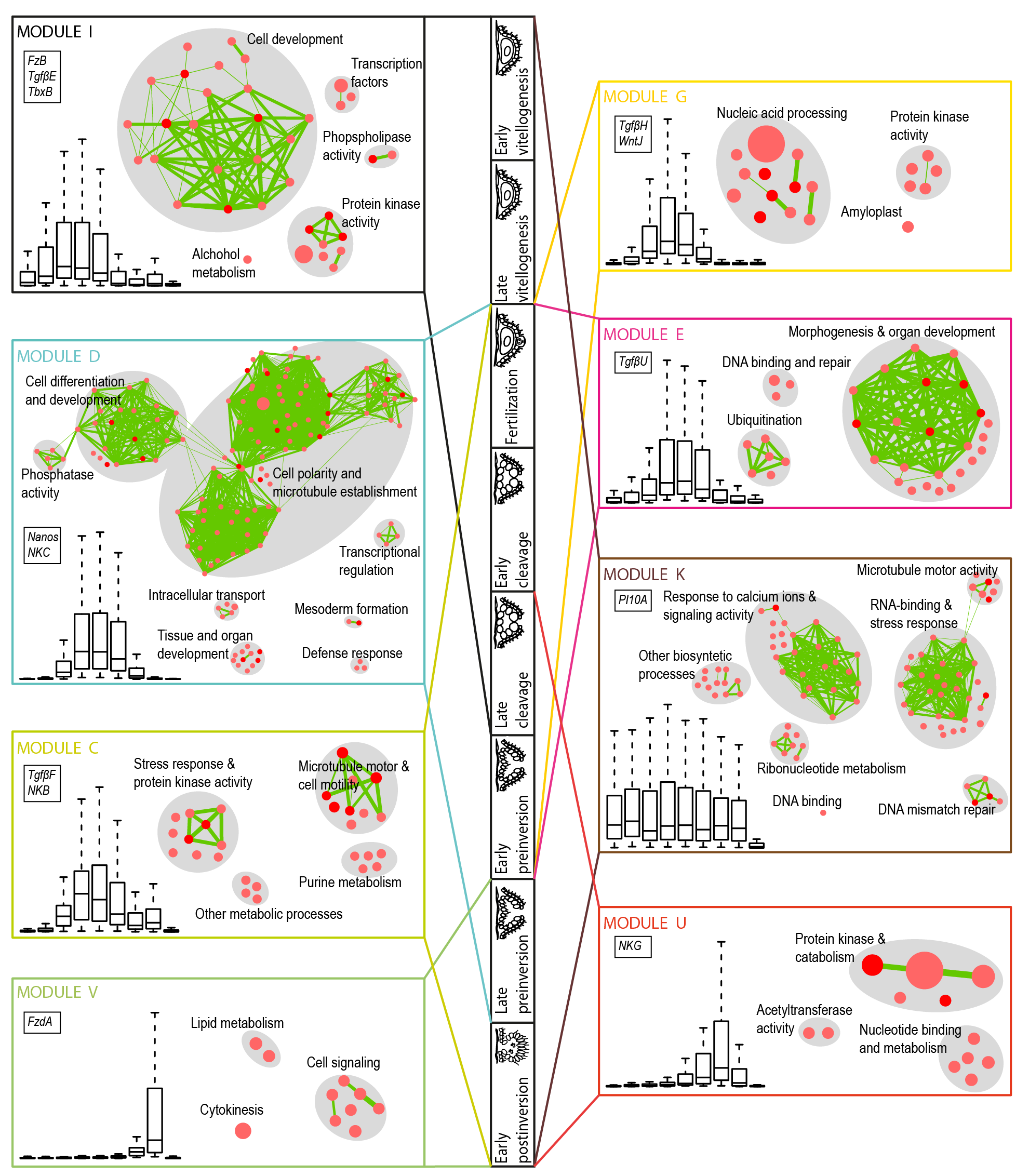

Supplement: GO-enrichment analysis of selected modules [file rspb20151746supp3.png]
